# *In Situ* Transformation of Tin Microparticles to Nanoparticles on Nanotextured Carbon Support Boosts the Efficiency of the Electrochemical CO_2_ Reduction

**DOI:** 10.1021/acsaem.4c02830

**Published:** 2025-02-10

**Authors:** Tom Burwell, Madasamy Thangamuthu, Elena Besley, Yifan Chen, Jasper Pyer, Jesum Alves Fernandes, Anabel E. Lanterna, Peter Licence, Gazi N. Aliev, Wolfgang Theis, Andrei N. Khlobystov

**Affiliations:** † School of Chemistry, University of Nottingham, University Park, Nottingham NG7 2RD, U.K.; ‡ Carbon Neutral Laboratory, University of Nottingham, Jubilee Campus, Nottingham NG7 2GT, U.K.; § School of Physics & Astronomy, University of Birmingham, Edgbaston B15 2TT, U.K.

**Keywords:** carbon nanofibers, nanotubes, tin nanoparticles, electrocatalysis, CO_2_ reduction reaction, formate production, electron microscopy

## Abstract

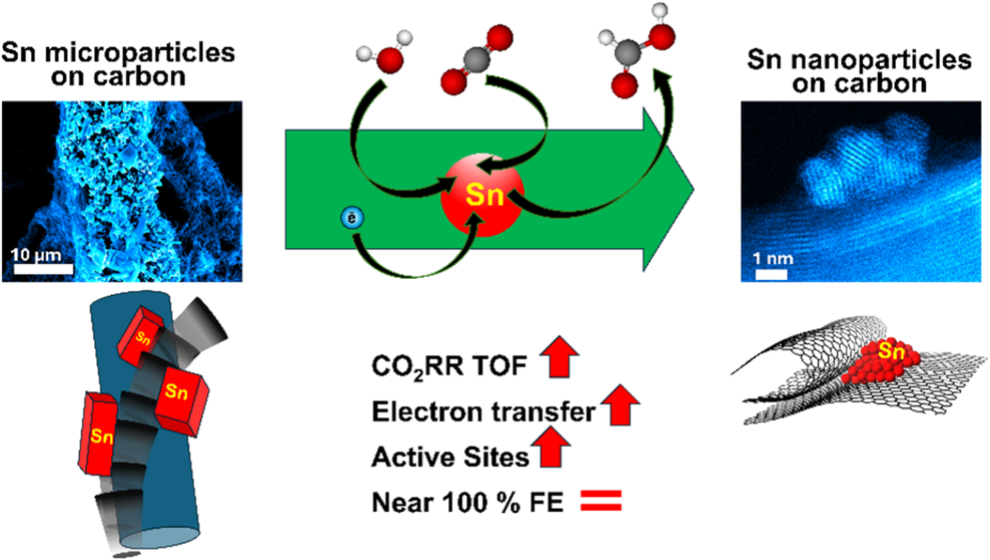

Developing sustainable, efficient catalysts for the electrocatalytic reduction of CO_2_ to valuable products remains a crucial challenge. Our research demonstrates that combining tin with nanostructured carbon support leads to a dynamic interface promoting the transformation of microparticles to nanoparticles directly during the reaction, significantly increasing the formate production up to 5.0 mol h^–1^ g^–1^, while maintaining nearly 100% selectivity. Correlative electrochemistry–electron microscopy analysis revealed that the catalyst undergoes an *in situ* self-optimization during CO_2_ electroreduction. It has been found that changes in the catalyst are caused by the breakdown of Sn particles driven by electrochemical reactions. The process of pulverization typically results in a decrease in the catalytic activity. However, when Sn particles are pulverized and reach approximately 3 nm in size on the surface of the nanotextured carbon support, the efficiency of the catalyst is maximized. This enhancement occurs because the *in situ*-formed Sn nanoparticles exhibit better compatibility with the nanotextured support. As a result, the number of electrocatalytically active sites significantly increases, leading to a reduction in charge transfer resistance by more than 2-fold and an improvement in reaction kinetics, which is evidenced by changes in the rate-determining step. Collectively, these factors contribute to a 3.6-fold increase in the catalyst’s activity while maintaining its selectivity for formate production.

## Introduction

Carbon dioxide is the main greenhouse gas contributing to environmental issues,^1^ but it can serve as a feedstock for producing valuable chemicals via the CO_2_ reduction reaction (CO_2_RR).^2^ Liquid fuels with high energy density, like ethanol and methanol, are highly desirable products of the CO_2_RR,^3,4^ and there is also demand for formic acid and formates, which find applications in chemical synthesis, antifreezing, and cooling processes.^5,6^ Several approaches, including photo, thermal, and electrocatalysis,^7−14^ can be used for the CO_2_RR, but the electrochemical route stands out due to its high efficiency,^15^ selectivity,^16^ and catalyst stability. Currently, electrocatalysts based on the main group elements such as tin (Sn),^17^ bismuth (Bi),^18,19^ and lead (Pb)^20,21^ lead CO_2_RR toward formate production. In particular, Sn has been studied in various forms, including nanoparticles (NPs),^22^ single atoms,^23^ and electrodeposited films.^24^ Electrodeposition of Sn micro or nanostructures offers a large surface area, resulting in high formate production rates.^25−27^ For example, An et al.^24^ demonstrated Faradic efficiency (FE) of 89% and stability over 9 h with moderate current density (−6 mA cm^–2^) using Sn on carbon paper. In contrast, Nguyen-Phan et al.^28^ reported excellent current densities (−73 mA cm^–2^) in a conventional H-cell for SnO_2_ NPs on a carbon black support but with a lower FE of 71%. A key challenge for the application of Sn in the CO_2_RR is the high overpotentials required for a high FE. To address this, Kempasiddaiah et al.^29^ reported a catalyst with the addition of copper (Cu) in a Cu–Sn alloy on reduced graphene oxide, achieving a FE of 80% at −0.69 V vs reversible hydrogen electrode (RHE) and current density of −12 mA cm^–2^. Furthermore, Rabiee et al.^30^ demonstrated another Cu–Sn system where Sn was electrodeposited onto Cu hollow fibers, resulting in formate FE of 78% and high current densities (−88 mA cm^–2^) but at a high overpotential (−1.2 V vs RHE). A bimetallic system of nanocrumpled Sn–Bi alloy, reported by Ren et al.,^31^ exhibited the current density of −39.5 mA cm^–2^ at −0.84 V vs RHE with high formate selectivity (96.4%) and stability over 160 h. Overall, current electrocatalysts based on Sn do not provide a balance between key performance parameters for the conversion of CO_2_ to formate. Improving one parameter often compromises another, as catalysts aiming for lower applied potential sacrifice current density, while those designed for high current densities suffer from high overpotential.^32,33^ Furthermore, complex catalyst synthesis may limit scaling up to industrial reactors.^34^ In this study, we present a catalyst made of Sn supported by carbon microfibers that have been modified with graphitized nanofibers (GNFs). The nanoscale texture of the GNFs plays a crucial role in the evolution of the Sn catalyst, which is transformed from micron-sized to nanometer-sized particles during electrochemical CO_2_RR. This transformation enables the catalyst to achieve a productivity of 5 mol h^–1^ g^–1^ with a FE near 100% for formate. This catalyst exhibits formate selectivity (>95%) across a wide overpotential range (−1.18 to −0.58 V vs RHE), and excellent stability, showing a 3.6-fold activity increase over 48 h. The excellent performance of the new catalyst is attributed to the self-tuning compatibility between the Sn particles and the carbon support, which maximizes the number of electrochemically active Sn atoms and enhances electron transfer in the CO_2_RR.

## Experimental Section

### Materials

GNFs were supplied by PyroGraf (PR-24-XT-HHT) with an iron content below 100 ppm. The GNFs were heat treated in air (300 °C) for 1 h to dry the carbon surface. Subsequently, Sn was electrodeposited on GNFs from anhydrous tin chloride (Fluka), sulfuric acid (95–97%), and sodium citrate tribasic dihydrate (Sigma-Aldrich).

### Electrochemical Characterization and Catalysis

All electrochemical experiments (LSV, EIS, and ECSA) were performed in a standard three-electrode cell (Ossila) using the Metrohm Autolab potentiostat (PGSTAT204 with FRAM32M module). The electrocatalyst deposited on PTFE-treated Toray carbon paper–060 (5 wt %) with a geometric surface area of 1 × 1.5 cm^2^ was used as the working electrode. Platinum (Pt) mesh and Ag/AgCl (3 M NaCl) were used as the counter and reference electrodes, respectively. All potentials recorded were converted to the reversible hydrogen electrode (RHE) and observed potentials are *iR* corrected using the Nernst equation, eq 1:
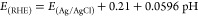
1For chronoamperometry, a Metrohm Autolab (PGSTAT302N) was used for all of the extended catalysis experiments. The gastight two-compartment H-cell (Ossila) fitted with a proton exchange membrane (Nafion117 – Sigma-Aldrich) was used for catalysis. Anode and cathode compartments were filled with 30 mL of electrolyte (0.1–2 M KHCO_3_). The Sn catalyst deposited working electrode was placed in the cathode compartment along with a reference electrode, and the counter electrode was placed in the anode compartment. CO_2_ was continuously bubbled in the cathode compartment with stirring (600 rpm). NMR samples were taken periodically every 30 min by disconnecting the gas exhaust tubing. Then, electrocatalysis was resumed after 10 min of CO_2_ purging to eliminate O_2_ and N_2_.

### XPS Data Collection and Analysis

XPS data was acquired with a Kratos AXIS Ultra DLD instrument using monochromated aluminum Kα emission at 120 W and a Thermo Scientific K-Alpha X-ray spectrometer with a monochromated aluminum source at 1486 eV. The energy used for these analyses was 12 kV under charge compensation conditions. High-resolution scans were acquired using a 20 eV pass energy and 0.1 eV step size and at different dwell times depending on signal-to-noise ratios. Casa XPS software (version 2.3.24) was used for data interpretation. Spectra were calibrated using the C 1s peak at 284.8 eV.

### Electrodeposition of Sn onto GNF

First, the ink was prepared by dispersing 1 mg of GNFs in 1 mL of ethanol using ultrasonication. Subsequently, three drops of 50 μL each were cast onto a carbon paper (CP) electrode with a geometric surface area of 1 × 1.5 cm^2^ and the electrode was allowed to dry at room temperature for 2 h. The resulting GNF-CP electrode was then placed in a three-electrode cell containing 40 mL of deionized water, 17 mM SnCl_2_, 68 mM sodium citrate, and 100 μL of 0.5 M sulfuric acid. To electrodeposit Sn onto the GNF support, a constant potential of −0.75 V vs RHE was applied, normalized to −0.294 C, with mild magnetic bar stirring (200 rpm). After the deposition, the electrode was cleaned by immersing it in DI water several times to remove any residual electrolyte.

### Product Analysis

Liquid products were analyzed using a Bruker AV(III) 500 MHz NMR with ^1^H solvent suppression (H_2_O) (Figure S1). An aliquot of the electrolyte (0.4 mL) was added to D_2_O (48 μL) and DMSO (40 μL from 4 mM concentration) as an internal standard. The product concentration was calculated using eq 2,^35^ and the Faradaic efficiency (FE) was calculated using eq 3.
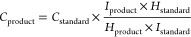
2where *C*_standard_, *I*_standard_, and *H*_standard_ are the concentration of the standard (0.4 mM), the integrated area of internal standard, and the number of hydrogen atoms present on the standard, respectively. The *C*_product_, *I*_product_, and *H*_product_ are the concentration of the product, area of integration of the product, and number of hydrogen atoms corresponding to the NMR peak, respectively.
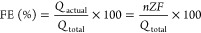
3where *Q*_total_ is the total charge passed, *Q*_actual_ is the amount of charge needed for *n* moles of product, *Z* is the number of electrons needed to form the product, and *F* is the Faraday constant.

## Results and Discussion

**Figure 1:**
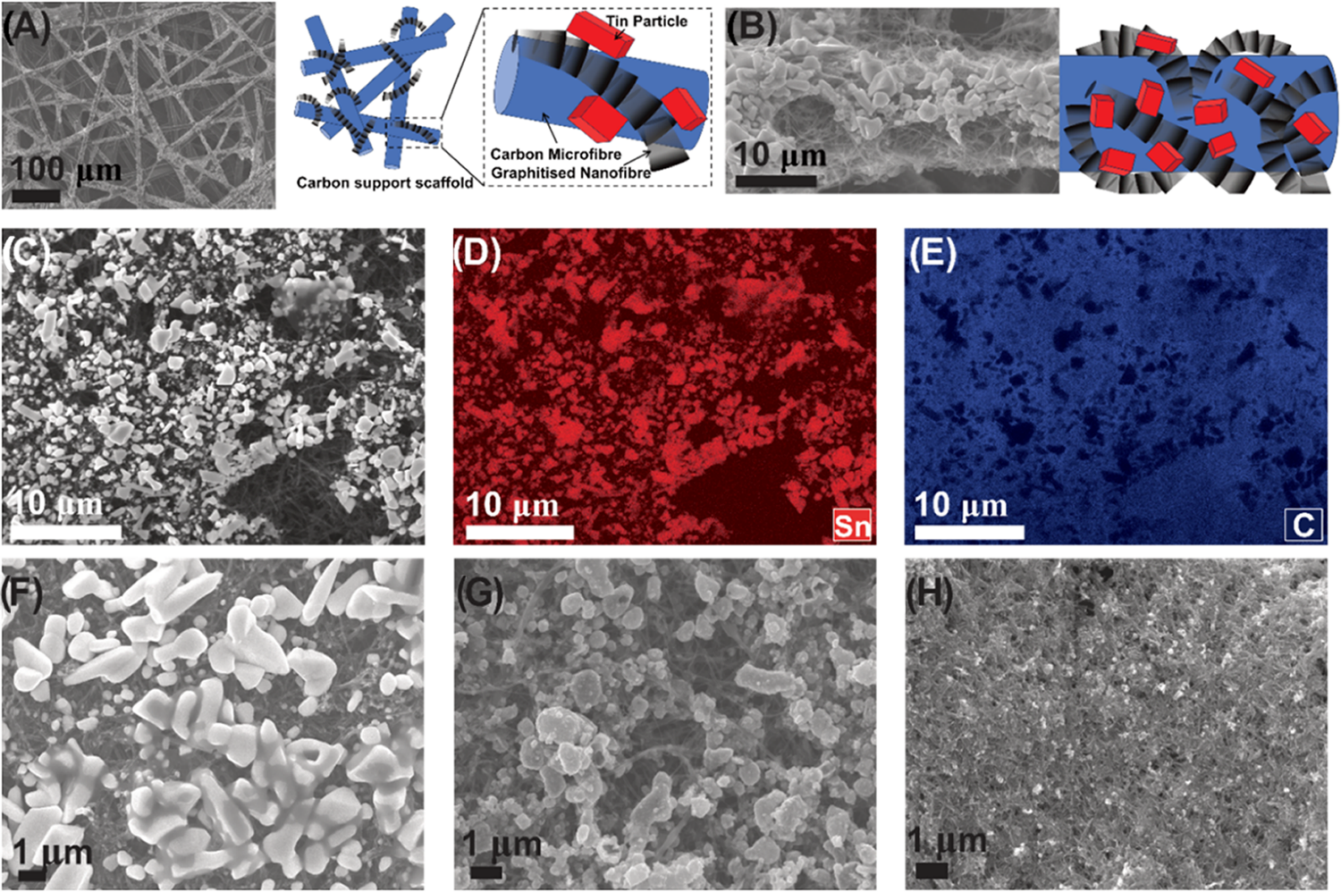
SEM images of electrodeposited Sn on GNFs. (A) Low magnification image of Sn deposited on GNFs with a schematic diagram of GNFs on carbon microfibers; (B) high-magnification image of cuboid-like structures with schematic illustrating GNFs on carbon microfibers; (C) low magnification image of Sn/GNF; (D) EDX map of Sn; (E) EDX map of panel (C); SEM images of Sn/GNF catalyst after (F) 2 h; (G) 24 h; and (H) 48 h of CO_2_RR in 1 M KHCO_3_.

Interactions and bonding of catalytic centers with support material play a significant role in determining catalysts’ activity, selectivity, and stability. GNFs possess excellent electrical conductivity and stability, which enable the use of transition metals in thermal,^36−40^ and electrochemical catalysis.^41,42^ In this study, we deposited GNFs with a diameter of 50–70 nm onto 5–10 μm carbon microfibers within a sheet of carbon paper (Figure 1A). Scanning electron microscopy (SEM) images show flexible GNF wrapping around the carbon fibers with the latter forming an open structure that can allow effective access to GNF as well as good electrical contact (Figure 1A,B). We chose to use electrodeposition to deposit Sn from an aqueous solution directly onto the working electrode (see Experimental Section). This method is easy and reproducible, making it suitable for mass production of the catalyst. However, it results in a nonuniform size distribution of the Sn particles. Sn microparticles (≥1 μm) are distributed along the network of GNFs, indicating the GNFs provide more favorable charge transfer, confirmed by electrochemical impedance spectroscopy (EIS) (Figure S2), and more favorable nucleation sites (Figure S3). This can be explained by the nanotextured surface of the GNFs providing more stable sites for metal atom adsorption and particle growth, which can lead to beneficial properties for the CO_2_RR.^43^

Energy-dispersive X-ray (EDX) spectroscopy mapping confirmed the electrodeposition of Sn (Figure 1C–E), demonstrating that the catalyst consists of isolated particles attached to the GNF.

**Figure 2:**
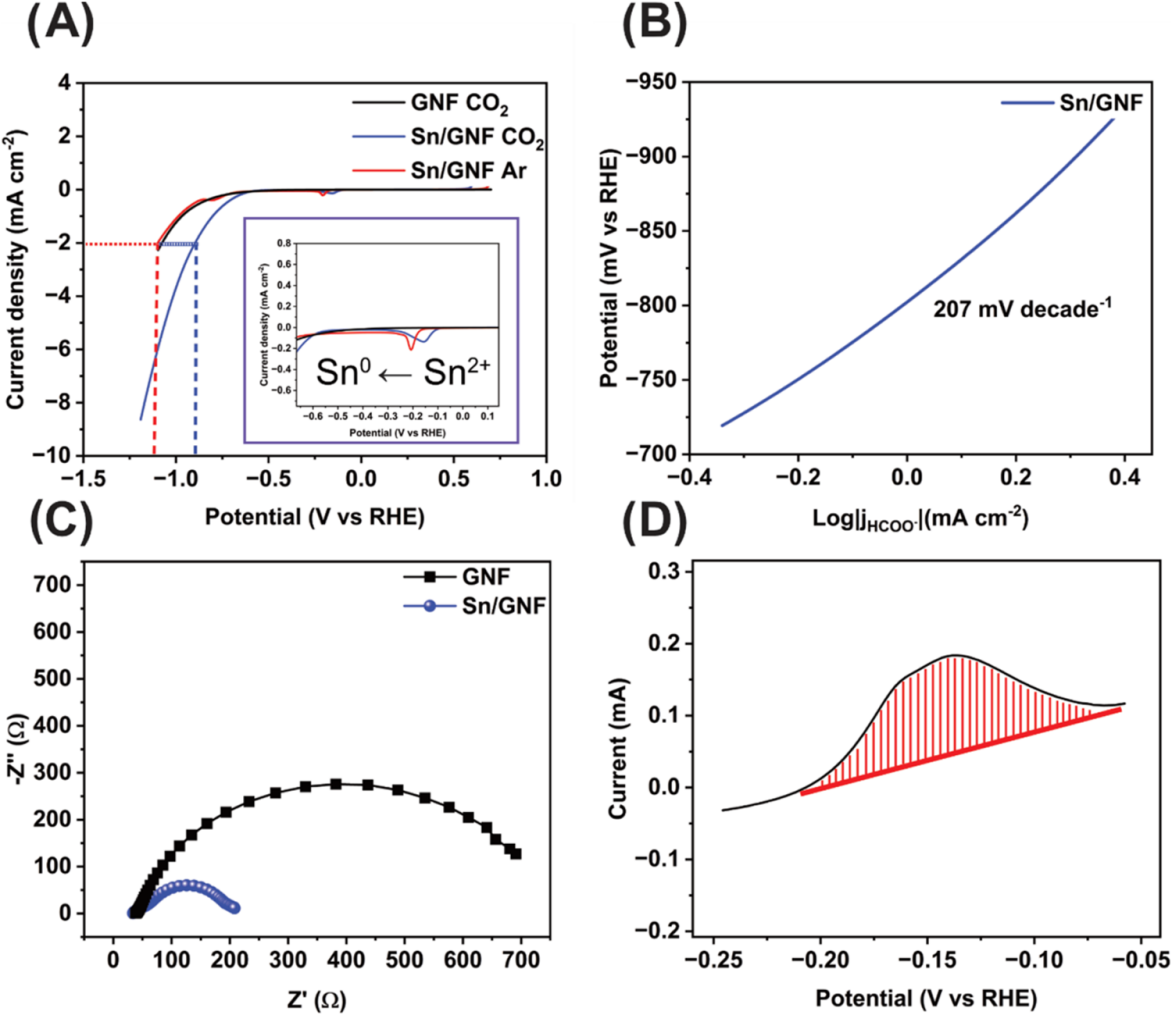
Electrochemical Characterization of Sn/GNF electrocatalyst: (A) LSV sweeping from +0.6 to −1.2 V vs RHE with a scan rate of 10 mV/s in 0.1 M KHCO_3_ saturated with CO_2_ (blue curve) and Ar (red curve), the onset potential indicated by a dashed line, and inset showing reduction of Sn^2+^ to Sn^0^; (B) Tafel plot of Sn/GNF extracted from (A); (C) Nyquist plot of Sn/GNF obtained in 0.1 M KHCO_3_ at a constant potential of −0.68 V vs RHE within the frequency range of 10 MHz to 0.01 Hz and amplitude of 10 mV_RMS_; and (D) integration of Sn^0^ to Sn^2+^ oxidation of tin to extract the number of surface active sites.

The electrochemical CO_2_RR activity of the Sn/GNF material has been studied by the linear sweep voltammetry (LSV) in 0.1 M KHCO_3_ electrolyte solution, showing a distinct reduction peak corresponding to the reduction of Sn^2+^ to Sn^0^.^44^ Additionally, from the LSV an increase in current density was observed in the presence of CO_2_ (Figure 2A). At–2 mA cm^–2^, the Sn/GNF shows the CO_2_RR onset potential of −0.89 V vs RHE, which is less than the onset potential observed in the presence of Ar (−1.10 V vs RHE), indicating a high CO_2_ reduction activity of the Sn/GNF catalyst. Then, we assessed the reaction kinetics of the CO_2_RR; the obtained Tafel slope value of 207 mV decade^–1^ (extracted from the LSV) (Figure 2B) demonstrated the slow kinetics. It indicates that the rate-determining step (RDS) must be controlled by diffusion of CO_2_ (Scheme S1, steps 1 to 1a), which agrees well with earlier reports.^45−48^ Furthermore, we measured the catalyst’s charge transfer resistance, showing that adding Sn to the GNF support reduces the charge transfer resistance from 702 to 208 Ω (Figure 2C), with an electrolyte resistance of 34 Ω. Finally, the number of electrochemically accessible Sn atoms in the catalyst material was calculated using cyclic voltammetry (CV) by integrating the area under a two-electron oxidation Sn^0^/Sn^2+^ peak (Figure 2D), revealing 3.09 × 10^18^ of Sn atoms (see Supporting Information for detailed calculation). Using this number, we calculated the turnover frequency (TOF) of 2.62 × 10^5^ h^–1^ for formate production at −0.98 V vs RHE in 0.1 M KHCO_3_.

**Figure 3:**
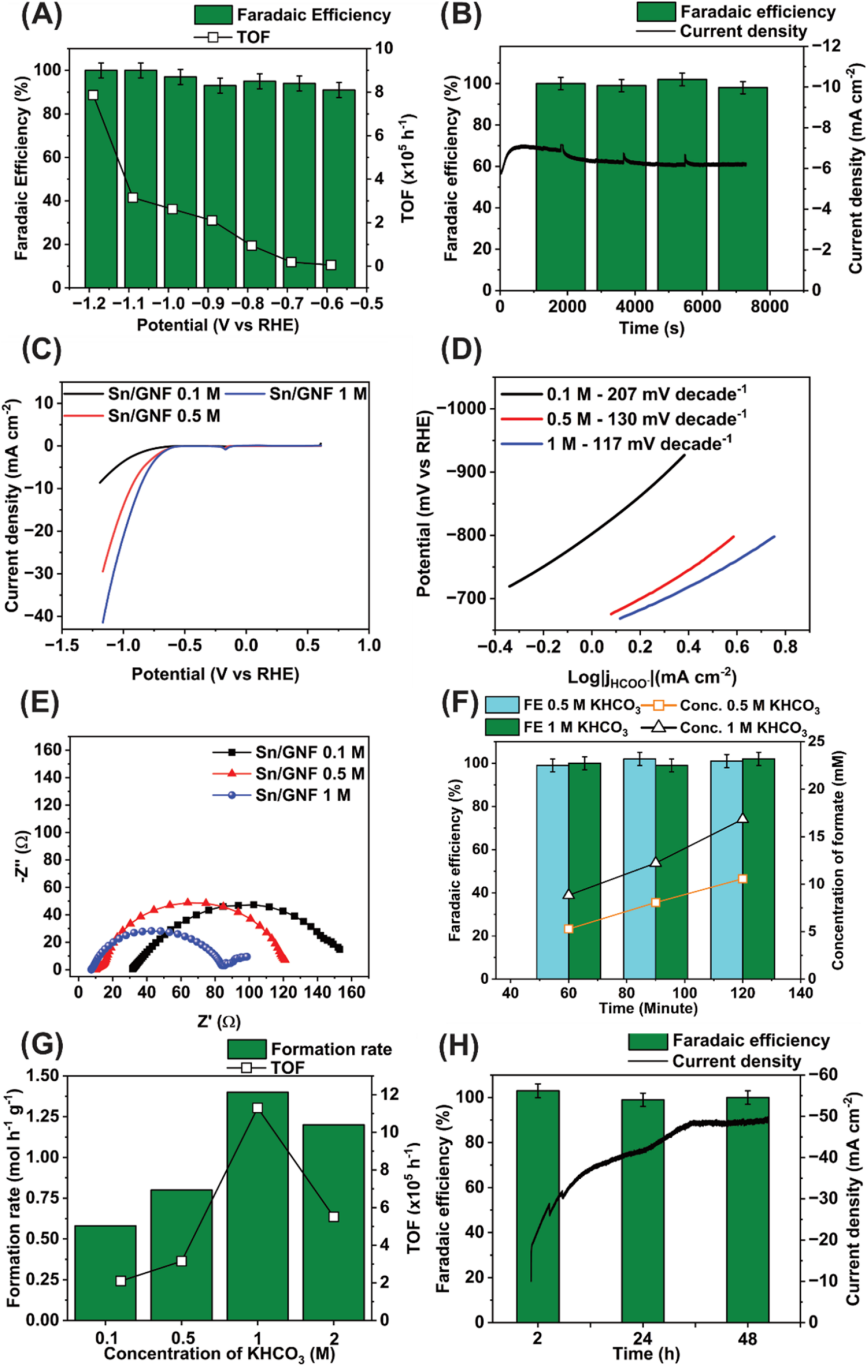
CO_2_RR Performance of Sn/GNF: (A) FE and TOF of Sn/GNF at potentials ranging from −1.18 to −0.59 V vs RHE in 0.1 M KHCO_3_; (B) 2-h stability test in 0.1 M KHCO_3_ at −0.98 V vs RHE and the corresponding FE and current density; (C) CO_2_RR activity of Sn/GNF in 0.1, 0.5, and 1 M of KHCO_3_ electrolyte solution while sweeping the potential from +0.6 to −1.19 V vs RHE with 10 mV/s scan rate; (D) Tafel slopes derived from panel (C); (E) Nyquist plot of Sn/GNF while varying electrolyte concentrations at a constant potential of −0.68 V vs RHE within a frequency range of 10 MHz to 0.01 Hz and an amplitude of 10 mV_RMS_; (F) comparison of the CO_2_RR activity observed in 0.5 and 1 M KHCO_3_ electrolyte over 2 h; (G) formate production rate and TOF obtained while increasing KHCO_3_ concentrations; and (H) 48-h stability test at −0.89 V vs RHE in 1 M KHCO_3_ and the corresponding modulus of current density (black curve) and FE of formate showing that activity of the catalyst increases with time while FE for formate remains stable.

The CO_2_RR activity of the present Sn/GNF catalyst over time was evaluated by using chronoamperometry in 0.1 M KHCO_3_ electrolyte. Interestingly, the liquid product formate with a FE of over 96% was observed while applying the potentials in the range of −0.8 to −1.2 V vs RHE (Figure 3A), with a production rate of 1.8 mol h^–1^ g^–1^ at −1.18 V. In the range of −0.88 to −0.58 V vs RHE, the TOF drops, but the FE remains high (>80%), indicating the excellent selectivity of the catalyst at said overpotentials. We have selected a medium overpotential (−0.98 V vs RHE) at which the catalyst is sufficiently productive (ca. 0.58 mol h^–1^ g^–1^) and near 100% selective for further investigation. After 2 h of catalysis, the FE of the CO_2_RR remains near 100% (Figure 3B), demonstrating that the Sn/GNF is stable. Then, the electrocatalyst’s CO_2_RR activity was tested with varying electrolyte KHCO_3_ concentrations from 0.1 to 2 M to increase the rate of formate production, partial current density, and CO_2_ concentration. The CO_2_RR current density increased by a factor of 3 from 0.1 to 0.5 M, with a further but modest increase in 1 M (Figure 3C) and achieved a maximum CO_2_RR current density of −41 mA cm^–2^ at −1.16 V vs RHE in 1 M KHCO_3_. The relationship between increasing current density and electrolyte concentration can also be observed in the Tafel plot, with a concomitant relationship between electrolyte concentration and decreasing gradient (Figure 3D). Therefore, as the concentration is increased, the RDS shifts from a diffusion-controlled process to the first electron reduction (eq 4, Scheme S1 step 3), which is evidenced by the obtained lower Tafel slope of 117 mV dec^–1^ for 1 M compared to the 0.5 M (130 mV dec^–1^), indicating the kinetics of the CO_2_RR has been significantly enhanced.^45^ We also observed a decrease in charge transfer resistance from 208 to 122 Ω, while increasing the electrolyte concentration from 0.1 to 0.5 M, respectively, and further to 83 Ω in 1 M solution (Figure 3E).
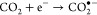
4
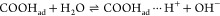
5

Figure 3F compares the FE and the formate production rate measured in 0.5 and 1 M electrolyte solutions. The FE remains stable over 2 h for both concentrations and shows no difference in selectivity. The TOF reaches a maximum of 1.13 × 10^6^ h^–1^ in 1 M KHCO_3_ as compared to 3.15 × 10^5^ h^–1^ in 0.5 M KHCO_3_. Increasing the electrolyte concentration from 0.1 to 1 M leads to a 5.6 times higher formate production rate. However, further increasing the electrolyte concentration to 2 M causes a decrease in the catalyst productivity (Figure 3G), which may be attributed to K^+^ ions blocking the active Sn sites, leading to the less available sites for CO_2_ adsorption.^49^ Overall, the highest catalyst productivity of 1.4 mol h^–1^ g^–1^ over 2 h is achieved in 1 M KHCO_3_ at −0.89 V vs RHE. Therefore, these conditions were taken forward for the long-term stability test of the electrocatalyst. Interestingly, the FE remains constant over 48 h during the long-term test; however, surprisingly, the current density has increased by a factor of 4.08 from −12 to −49 mA cm^–2^ (Figure 3H, black curve). Such a drastic change in the catalyst activity, while maintaining a high selectivity, may indicate an increase in the number of active centers during the reaction.

To explore the mechanisms behind the enhancement of catalyst activity, we studied the changes in Sn/GNF using nanoscale imaging and analyzed structural changes that correlate with the evolution of the catalyst. SEM imaging of the Sn/GNF after 2 h shows the process of smoothing edges of the cuboid Sn particles continues (Figures 1F and S4). However, after 24- and 48 h of CO_2_RR, Sn particles become significantly smaller, less than a tenth of the original size (Figures 1G,H and S5), and become more uniform and intimately interspersed with the GNF support. As the leaching of Sn to the electrolyte occurs at only about 5% (measured by inductively coupled plasma-optical emission spectroscopy (ICP-OES)) over 48 h (Table S1), the process of reducing particle size is likely driven by pulverization, i.e., the reduction of the Sn oxide layer, creating defects in the crystalline structure, which induces stress and insertion of hydrogen ions, converting to H_2_ in the defects to break apart particles.^50,51^

**Figure 4:**
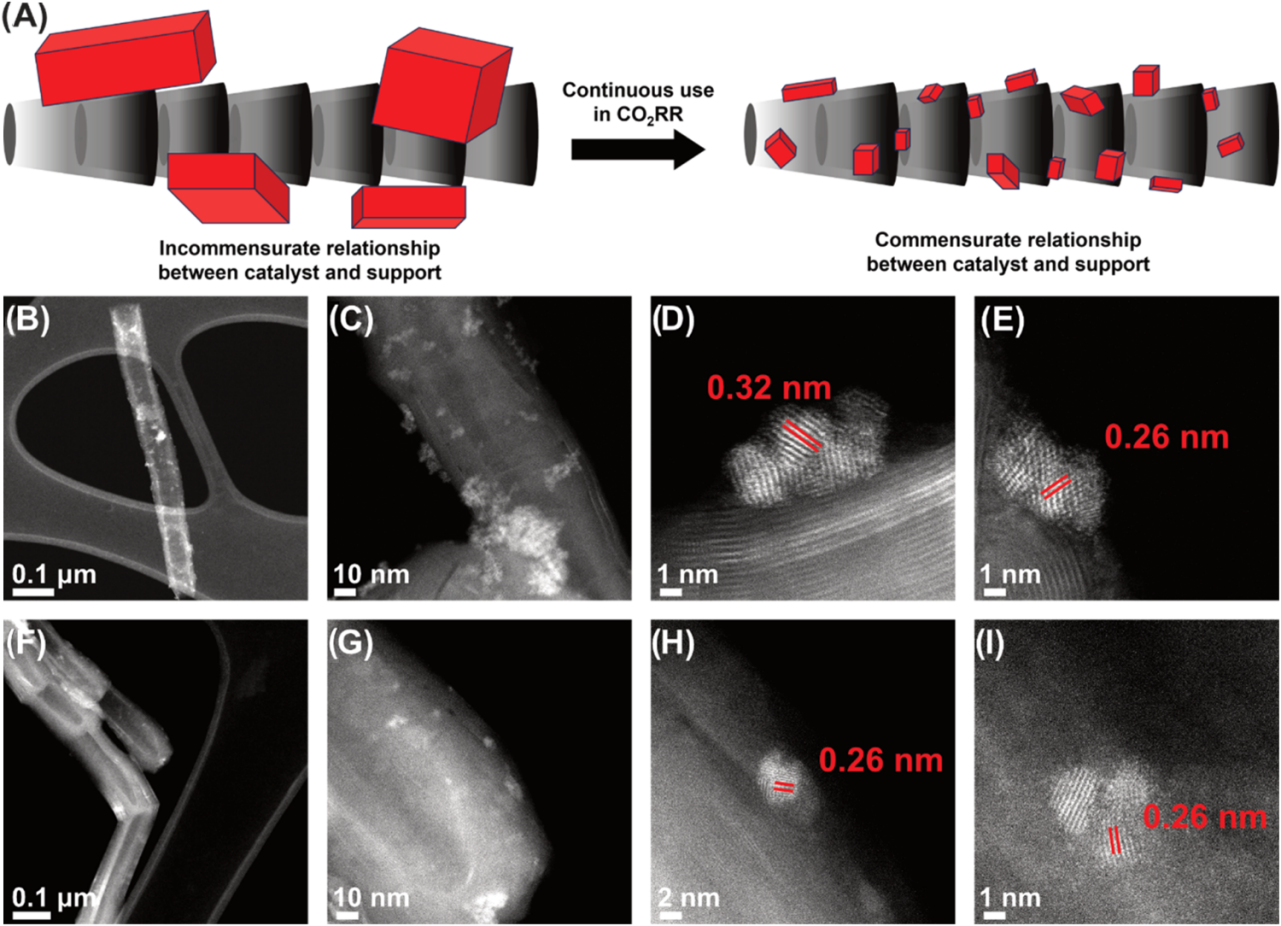
(A) Schematic representation of pulverization of Sn during the CO_2_RR. Darkfield AC-STEM images of Sn on GNFs after catalysis (B–E) for 24 h; and (F–I) after 48 h. (B) Low magnification of several particles of Sn on GNFs; (C) illustrating clusters of Sn particles; (D) graphitic layers of the GNF in close contact with small NPs of Sn; (E) Sn NP located on the step-edge of the GNF; (F) low magnification showing barely visible clusters of Sn particles; (G) high magnification of NPs on the tip of the GNF; (H) a NP of Sn with close contact between the Sn and the GNF; and (I) a small cluster of Sn NPs located on the GNF.

To elucidate the electrocatalyst changes in the nanoscale, we take advantage of the fact that GNFs are sufficiently thin and can be penetrated by the electron beam of the transmission electron microscope (TEM) to allow atomic-scale analysis of the catalytic sites involved in the reaction.^43^ We imaged the catalyst after 24 and 48 h of chronoamperometry by aberration-corrected scanning transmission electron microscopy (AC-STEM) alongside LSV, EIS, and Tafel analysis to assess Sn evolution during the reaction. The postreaction AC-STEM imaging after 24 h reveals the presence of 3–5 nm particles on the GNF surface, strongly suggesting the transformation of Sn microparticles during the reaction (Figure 4B–E), with some larger clusters of NPs also present. After 48 h, the population of small particles further increases (Figure 4F–I); in addition, more well-defined, 2–3 nm particles emerged (Figure 4G–I). High-magnification AC-STEM images show that the NPs have a crystalline structure, with their atomic planes existing in direct contact with the carbon layers of the GNF (Figure 4D,E,H,I). After 24 and 48 h of reaction, the size of the catalytic NPs becomes commensurate with the diameter of GNF and nanoscale structural features of GNF (Figure 4D). The lattice observed in high-magnification AC-STEM images may correspond to the *P42/mnm* phase of SnO_2_ oriented along the [1 1̅ 1̅] zone axis (Figure S6), and the lattice spacings of 0.26 and 0.32 nm correspond to (101) and (110) planes (Figure 4D,E,H,I), respectively. Further analysis by XPS supports the presence of SnO_2_ before and after catalysis. The as-prepared catalyst contains Sn^0^ (∼7%) and the rest Sn^4+^ as shown by XPS peaks at 485.3 and 487.3 eV, respectively, whereas after 24 and 48 h of CO_2_RR, no Sn^0^ is found but much rather Sn^2+^ observed at 486.5 eV and Sn^4+^ at 487.1 eV, respectively (Figure S7).^52^

The overall effect of the *in situ* pulverization of Sn microparticles results in a decrease in the size to 3 nm after 48 h (Figure 4A) accompanied by partial leaching of Sn into the electrolyte (Table S1). Afterward, the size of the Sn particles matches that of the GNFs, resulting in the formation of nanoparticles. This enhances contact between the Sn and the GNF, improving the catalyst’s stability and activity. In our measurements, the *in situ* pulverized Sn/GNF catalyst exhibits a higher rate of formate production, enhancing the charge transfer and improving the kinetics of the CO_2_RR while maintaining near 100% FE. This is in stark contrast to previous works, such as by Wu et al. and Kim et al., where a pulverization process worsens the CO_2_RR activity and enhances the parasitic hydrogen evolution reaction (HER).^50,51,53^ Our research indicates that if the size of the nanoparticles generated during the pulverization process matches the nanoscale features of the support, this previously unwanted occurrence during electrocatalysis can be utilized to enhance the catalytic activity without compromising selectivity.

**Figure 5:**
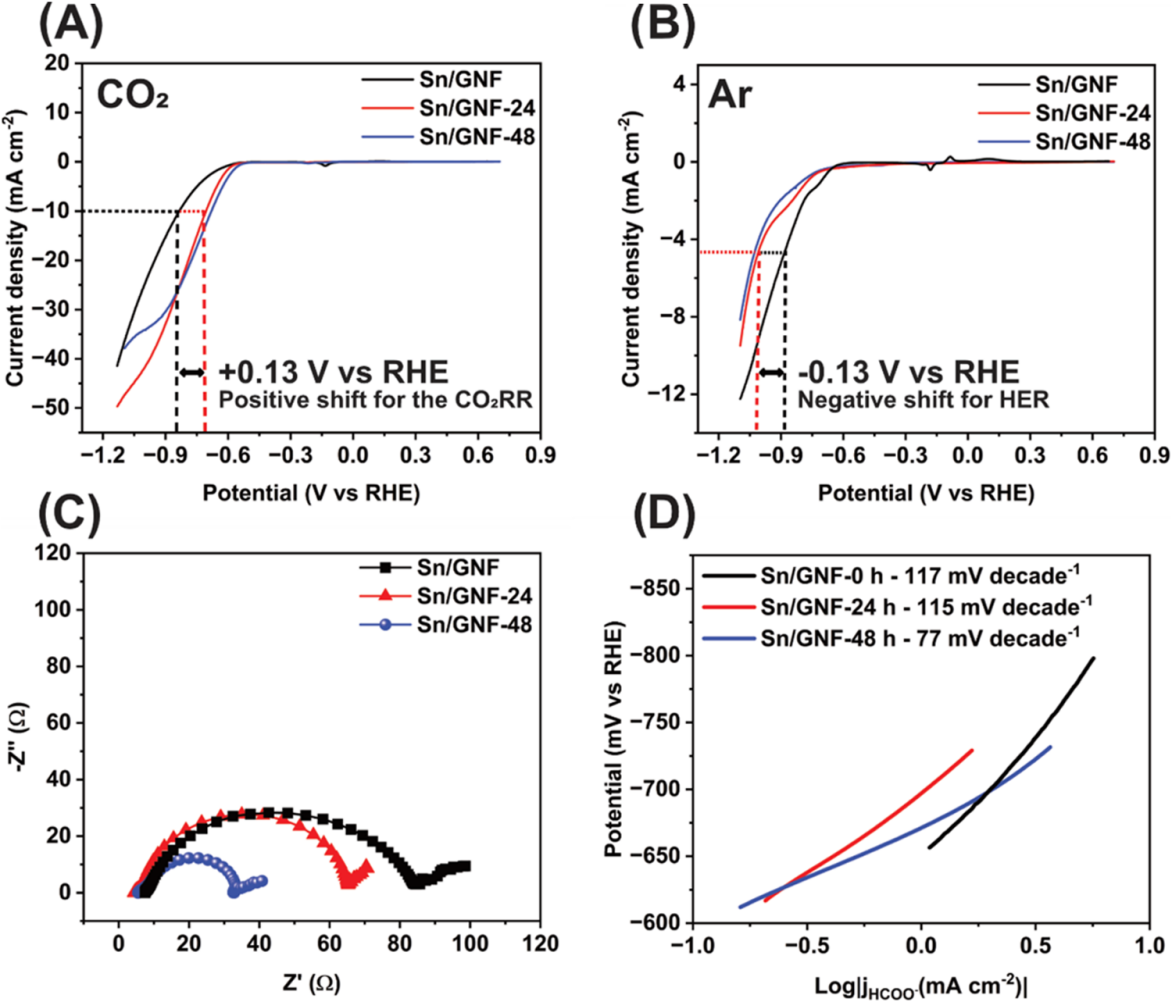
(A) LSV comparison of fresh catalyst vs postreaction after 24 and 48 h of CO_2_RR, in 1 M KHCO_3_ sweeping the potential from +0.6 to −1.19 V vs RHE with a scan rate of 10 mV/s in CO_2_ saturated condition with onset taken at −10 mA cm^–2^; (B) under Ar-saturated condition with onset taken at −5 mA cm^–2^; (C) Nyquist plot comparison of postreaction catalyst obtained under constant potential of −0.68 V vs RHE within the frequency range from 10 MHz to 0.01 Hz; and (D) Tafel plots derived from the LSV of pre and post reaction of the Sn/GNF catalyst.

The changes in the catalyst particle size during the CO_2_ reduction reaction increased the partial current density (Figure 5A,B). The overpotential for CO_2_ reduction is reduced by +0.13 V vs RHE (from −0.83 to −0.70 V vs RHE) for the catalysts after 24- and 48 h reactions compared to the fresh catalyst. The CO_2_RR current density has effectively doubled from −14 to −32 and −29 mA cm^–2^ for the 24- and 48 h, respectively, at −0.89 V vs RHE (Figure 5A). Simultaneously, control experiments with Ar-saturated solutions show a high overpotential for water reduction (−0.89 to −1.02 V vs RHE) (Figure 5B). This indicates that Sn on GNF significantly increased its activity for the CO_2_RR and simultaneously decreased the level of the parasitic HER process. This is supported by EIS, illustrating an improvement in charge transfer as a function of time (Figure 5C). The charge transfer resistance decreased from 80 (fresh catalyst) to 65 Ω after 24 h and to 32 Ω after 48 h. Importantly, the number of electrochemically accessible sites in the catalyst increased from 3.09 × 10^18^ to 4.20 × 10^18^ after 24 h and further increased to 2.88 × 10^19^ after 48 h (Figures 1G,H and S8), allowing calculation of TOF after 48 h as 0.44 × 10^6^ h^–1^, and productivity reaching 5.0 mol h^–1^ g^–1^. The Tafel plot after 24 h shows a similar kinetics as the fresh catalyst in 1 M KHCO_3_ and maintains the same RDS (eq 4, Scheme S1 step 3), but after 48 h, the reaction kinetics is improved (Figure 5D, Scheme S1 step 2a) which manifests in the change of RDS (eq 5).^45^ This confirms that the *in situ* decrease in particle size improves charge transfer, kinetics, and partial current density for formate production in our experiments without requiring any special preparation of Sn NPs employed in previous works.^28,54−57^

## Conclusions

It is usual for the catalyst performance to decline during the reaction through various deactivation mechanisms. However, we demonstrate here that the Sn/GNF catalyst increases in partial current density for formate by a factor of 3.6 while maintaining near 100% selectivity for formate over 48 h of continuous usage in CO_2_RR. Our correlative electrochemistry and electron microscopy analysis approach reveals the nanoscale mechanisms behind this phenomenon. We showed that decreasing the size of the Sn catalytic centers during CO_2_RR promotes more effective contact with the carbon nanofiber support, which lowers the charge transfer resistance and simultaneously boosts the number of active centers by a factor of 10 over 48 h. These *in situ* structural and electrochemical improvements in Sn/GNF lead to faster reaction kinetics, hence higher catalyst productivity, in stark contrast to previous works in which such an *in situ* process worsened the catalyst’s performance. As such, our self-refining Sn/GNF catalyst under the reaction conditions displayed selectivity reaching nearly 100% FE for formate and one of the highest activities reported to date (Table S2 and Figure S9). Combined with the excellent stability of the catalyst, our work demonstrated Sn supported on a nanotextured conducting GNF surface as a highly selective and simple-to-make catalyst that requires minimal solvents and no use of precious metals.

Selecting the right catalyst support surface is crucial when designing an electrocatalyst. This is exemplified by the nanotextured support of the GNF, which helps overcome the challenges of pulverization or Ostwald ripening, often leading to catalyst degradation. In stark contrast to our findings, previous works reported such a pulverization process leading to worsening the CO_2_RR activity and enhancement of the parasitic HER.^50,51,53^ Our work bucks the trend, showing that this previously undesirable phenomenon occurring during electrocatalysis can in fact be harnessed to increase the catalytic activity without any loss of selectivity. We have shown that by matching the size of catalytic nanoparticles dynamically formed during the CO_2_RR not only can prevent the degradation of catalytic properties but also can boost the activity due to more facile charge transfer, while fully maintaining the catalyst selectivity at nearly 100%.

## Supporting Information Available

Scheme S1. CO_2_RR mechanism. Figure S1. Nyquist plots. Figure S2. SEM images of the catalyst. Figure S3. ^1^H NMR spectra of products. Figure S4. SEM images of the catalyst after 2 h CO_2_RR. Figure S5. SEM images of the catalyst after 24 h CO_2_RR. Figure S6. AC-STEM images of the catalyst after 2 h CO_2_RR. Figure S7. XPS of the catalyst after 24 and 48 h CO_2_RR. Figure S8. CV of the catalyst after 24 and 48 h CO_2_RR with surface active sites. Figure S9. Comparison of catalyst’s Faradic efficiency and current density to the literature data. Table S1. ICP-OES measurements for Sn in the electrolyte. Table S2. Comparison of key characteristics of similar catalysts reported in the literature. Equation S1. Calculation of turnover frequency. (PDF)
