# Supplementary material for: In Situ Transformation of Tin Microparticles to Nanoparticles on Nanotextured Carbon Support Boosts the Efficiency of the Electrochemical CO2 Reduction
Source: ACS Appl Energy Mater. 2025 Feb 10;8(4):2281–90. doi: 10.1021/acsaem.4c02830 (PMC11863182; doi:10.1021/acsaem.4c02830)
Supplement: Supplementary file 1 — ae4c02830_si_001.pdf [file ae4c02830_si_001.pdf]

Supporting information for

In situ transformation of tin microparticles to nanoparticles on nanotextured carbon support boosts the efficiency of the electrochemical CO_2 reduction

Tom Burwell,[1] Madasamy Thangamuthu,[1] Elena Besley,[1] Yifan Chen,[1] Jasper Pyer,[1] Jesum Alves Fernandes,[1], Anabel E. Lanterna,[1] Peter Licence,[2] Gazi N. Aliev,[3] Wolfgang Theis,[3] and Andrei N. Khlobystov[1]*

[1] T. Burwell, M. Thangamuthu, E. Besley, Y. Chen, J. Pyer, J. Alves Fernandes, A. E. Lanterna, A. N. Khlobystov.
School of Chemistry
University of Nottingham
Nottingham, University Park, NG7 2RD, UK

[2] P. Licence.
Carbon Neutral Laboratory
University of Nottingham
Nottingham, Jubilee Campus, NG7 2GT, UK

[3] G. N. Aliev, W. Theis.
School of Physics & Astronomy
University of Birmingham, Edgbaston
B15 2TT, UK

*Andrei.Khlobystov@nottingham.ac.uk

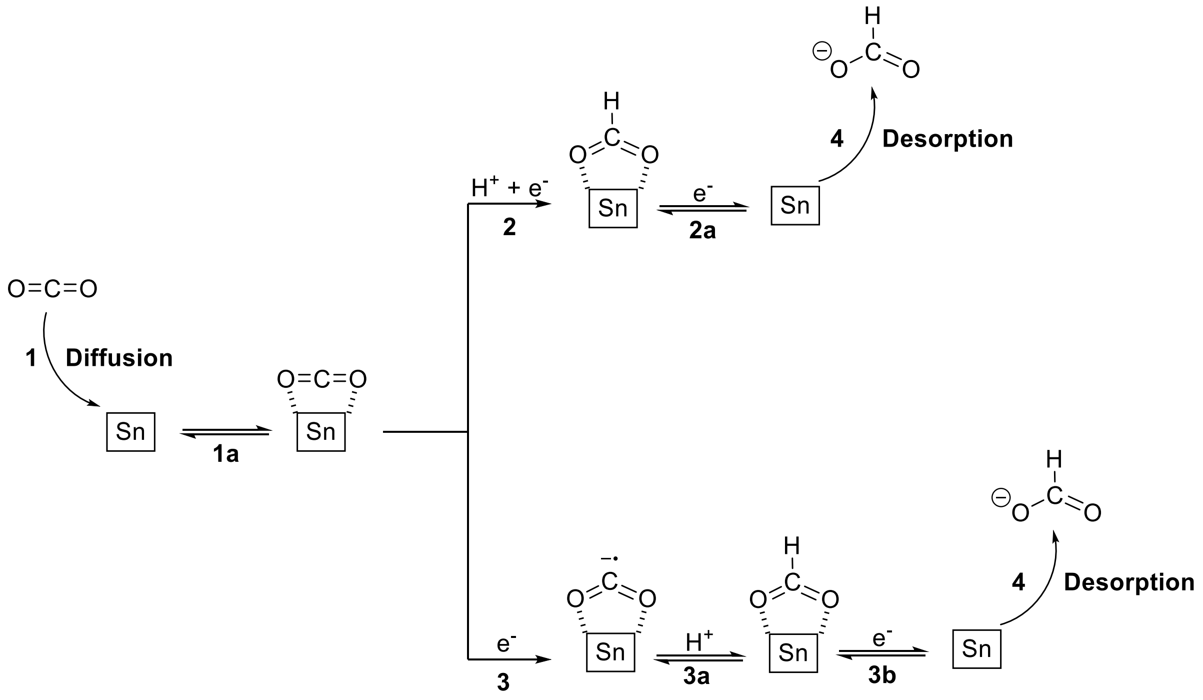

Scheme S1. Potential routes for the electrochemical reduction of CO_2 to formate

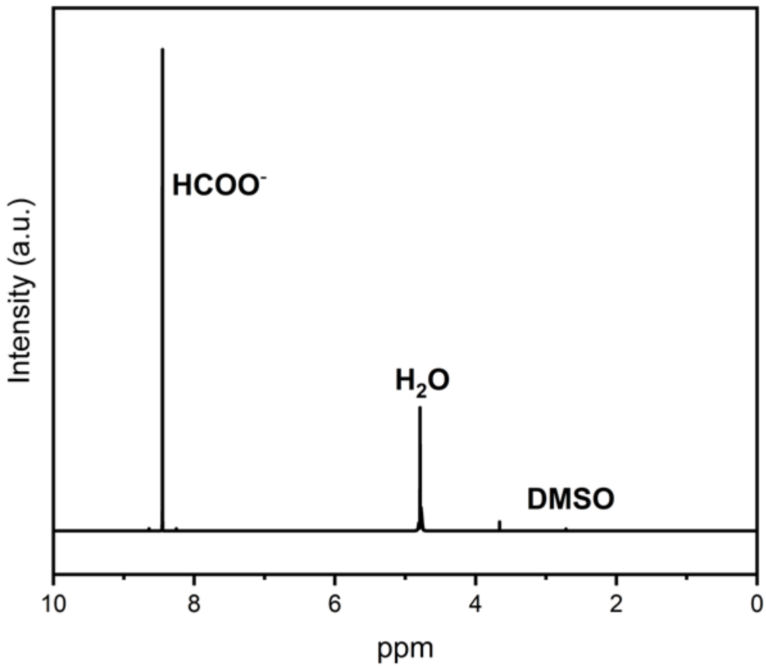

Figure S1. H_2O suppression ^1H NMR spectrum after 48 hours at -0.89 V vs RHE in 1 M $KHCO_3$.

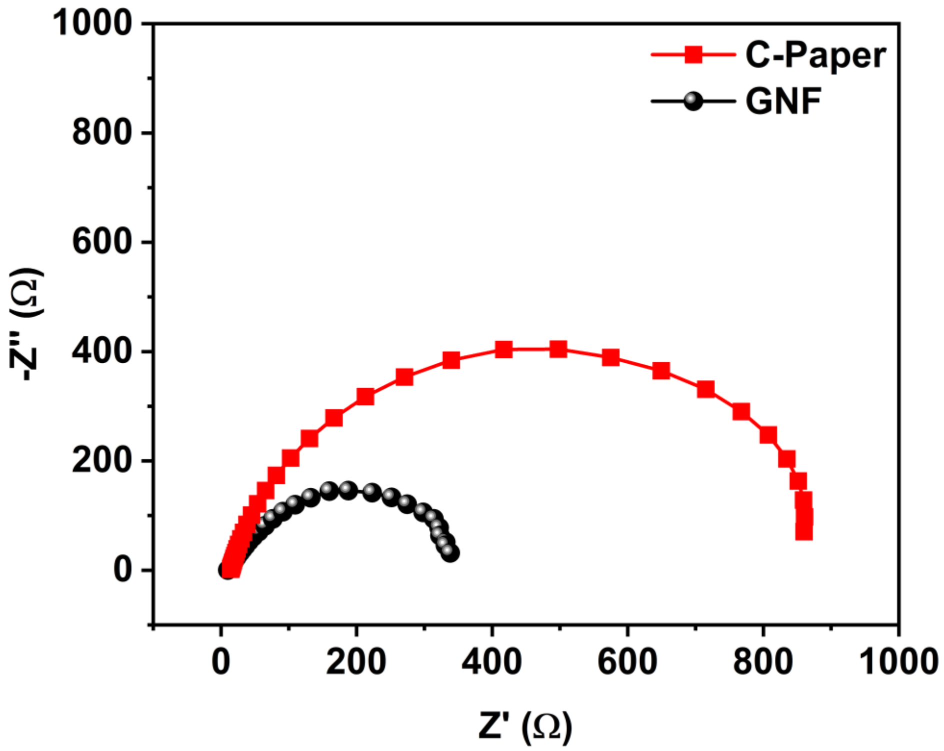

Figure S2. Nyquist plots of carbon paper with (black circle) and without (red square) GNFs deposition obtained in 1 M $KHCO_3$ at a constant potential of -0.68 V vs RHE within the frequency range from 10 kHz to 0.01 Hz and amplitude of 10 mV_{RMS}.

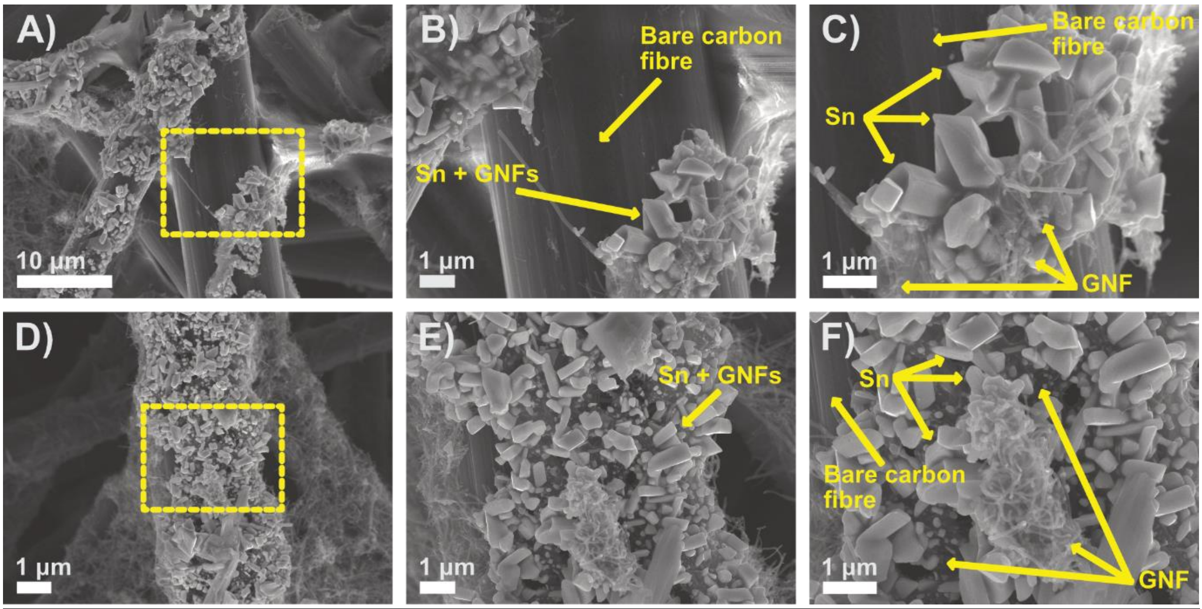

Figure S3. SEM images of electrodeposited Sn on GNFs coated carbon paper, (A, D) Low magnification of carbon microfibers show the areas free of GNFs remain free of Sn particles; (B, C, E, F) Zooming in the area of Sn nanoparticles reveals that they are connected with GNFs.

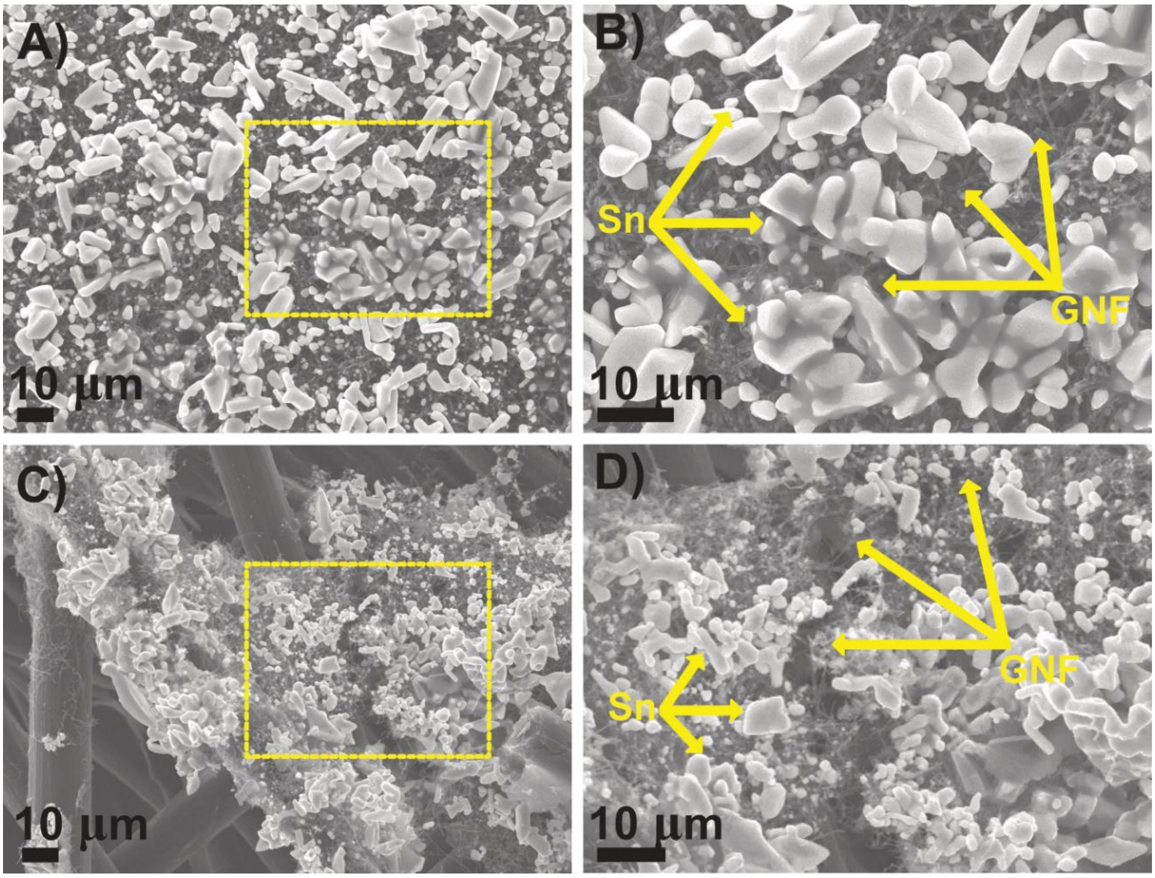

Figure S4. SEM images of electrodeposited Sn after 2 hours of electrocatalysis at -0.98 V vs RHE in 0.1 M $KHCO_3$. (A, C) Low magnification images of Sn anchored on mats of GNFs deposited on carbon paper; (B, D) High magnification image of Sn particles with smoothed edges attached to GNFs which can be seen on the background.

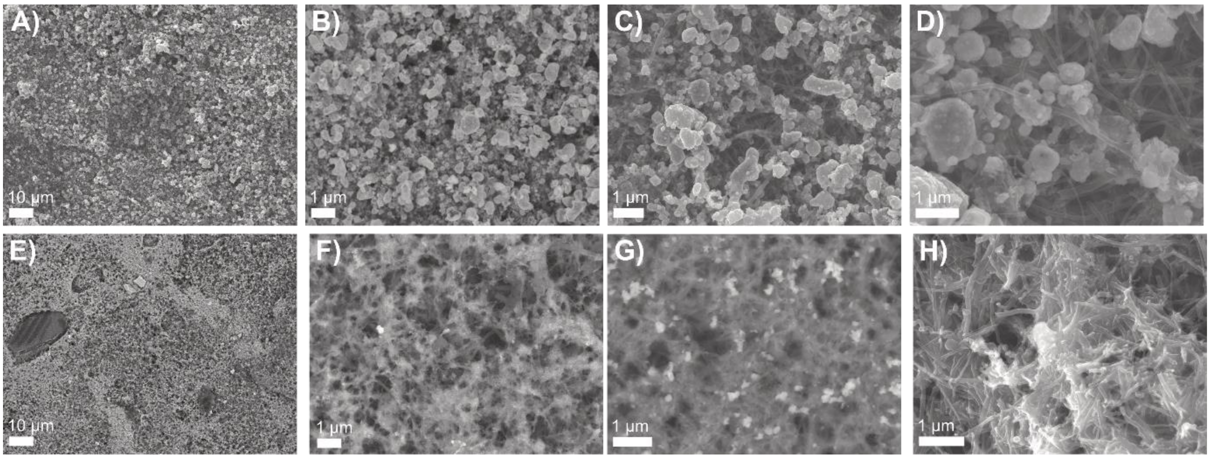

Figure S5. SEM images of electrodeposited Sn on GNFs on carbon paper after 24 hours (A-D) and after 48 hours (E-H) electrochemical CO_2RR in 1 M $KHCO_3$ at -0.89 V vs RHE.

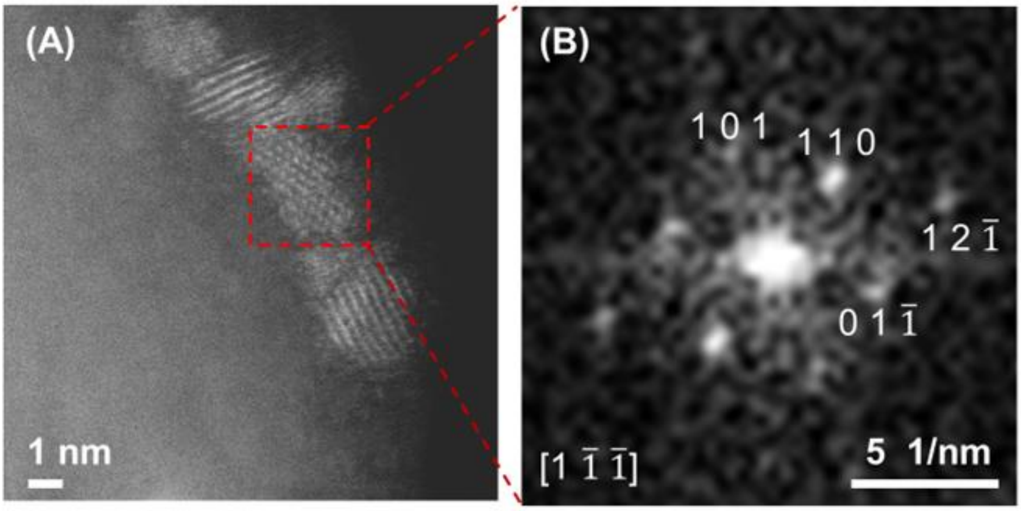

Figure S6. Darkfield AC-STEM image of Sn on GNFs after 24 hours CO_2RR in 1 M $KHCO_3$ at -0.89 V vs RHE and corresponding fast Fourier transform.

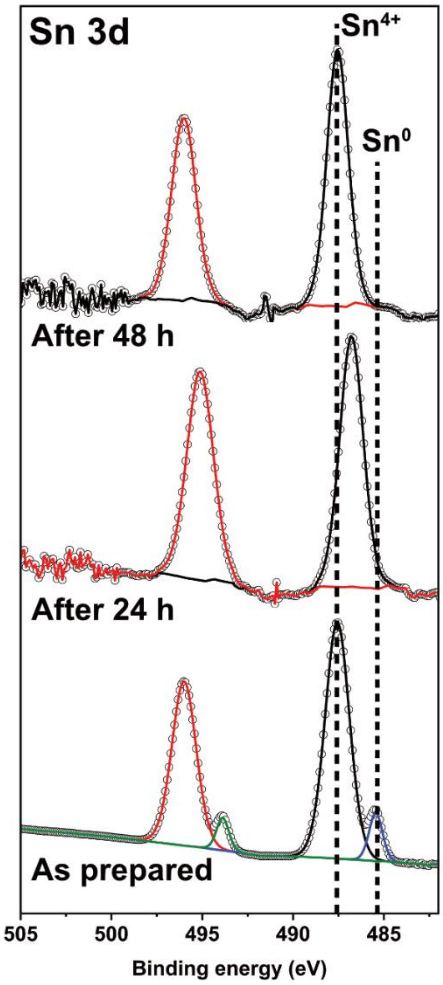

Figure S7. XPS of the Sn/GNF catalyst before reaction, after 24 hours and after 48 hours, red fitting for $3d_{3/2}$, black fitting for $3d_{5/2}$ (Sn^{4+}/Sn^{2+}) and blue fitting for Sn^0.

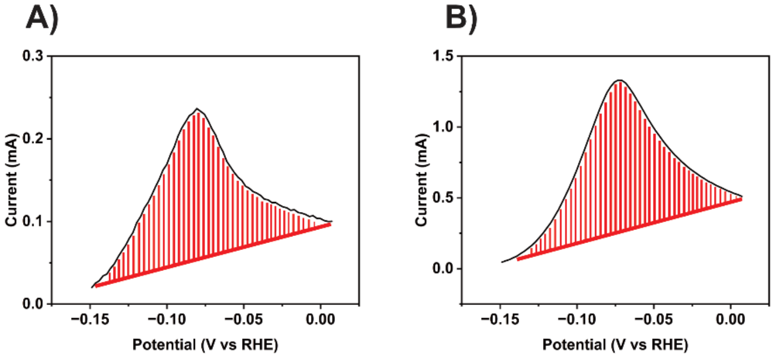

Catalyst	Surface active sites
Sn/GNF fresh	3.09 x10^{18}
Sn/GNF 24 h	4.20 x10^{18}
Sn/GNF 48 h	2.88 x10^{19}

Figure S8. CV post-reaction oxidation of Sn^0 to Sn^{2+} after A) 24 hours and B) 48 hours with a table showing surface active sites of Sn extracted from the oxidation peak.

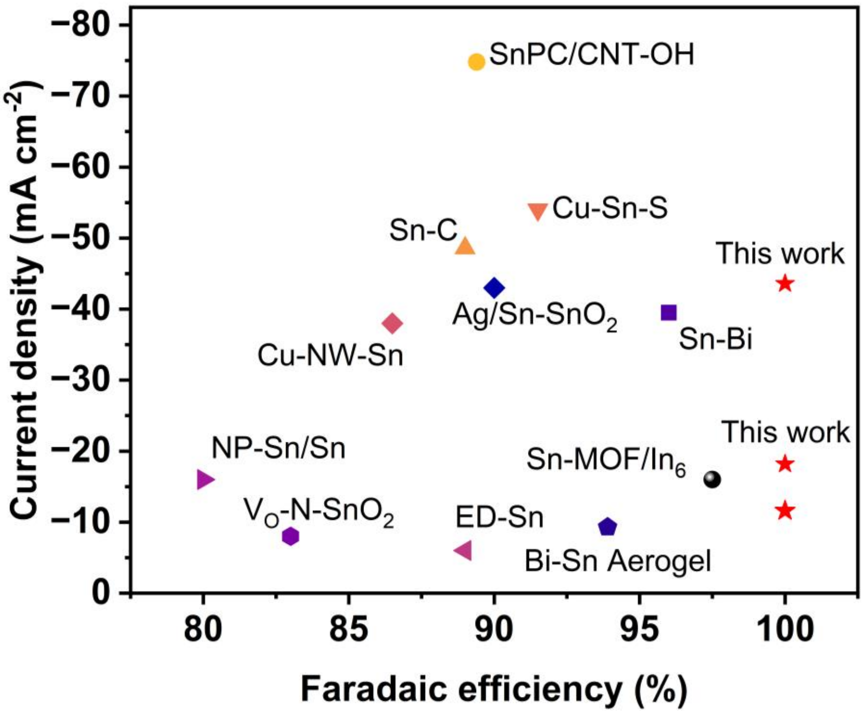

Figure S9. Literature comparisons to this work, see table 1 for value comparisons.

Table S1. ICP-OES results of the amount of Sn leaching into the electrolyte with a total starting loading of 180 µg of Sn

Catalyst - Hour	Mass of leached Sn (µg)	Sn remaining on the electrode (%)
Sn/GNF - 0	0	100
Sn/GNF – 12	5	97.2
Sn/GNF – 24	8	95.5
Sn/GNF – 48	10	94.4

Table S2. Literature comparison of Sn catalysts used for the electrochemical CO_2RR.

Catalyst	Concentration & electrolyte (M)	Current density (mA cm^{-2})	Applied Potential (V vs RHE)	Faradaic efficiency for formate (%)	Turnover frequency (h^{-1})
Sn/GNF*	0.1 $KHCO_3$	-11.6	-0.98	~100 ±5	2.65×10^5
Sn/GNF*	0.5 $KHCO_3$	-18.2	-0.95	~100 ±5	3.15×10^5
Sn/GNF* †	1.0 $KHCO_3$	-43.6	-0.89	~100 ±5	1.13×10^6
SnPC/CNT-OH	0.5 $KHCO_3$	-74.8	-1.0	89.4[1]	N/A
Sn-C cloth	0.5 $NaHCO_3$	-48.6	-0.88	87±2[2]	N/A
Cu-Sn-S	0.5 $KHCO_3$	-54.9	-1.2	91.5[3]	N/A
Cu-NW-Sn	0.1 $KHCO_3$	-38.0	-1.2	86.8[4]	N/A
Sn-CP	0.1 $KHCO_3$	-6.0	-1.1	89[5]	N/A
NP-SN/Sn	0.5 $NaHCO_3$	-16.0	-1.1	80[6]	N/A
V_o-rich N-SnO_2	0.1 $KHCO_3$	-8.0	-0.9	83[7]	N/A
Crumple induced Sn-Bi	0.5 $KHCO_3$	-40.0	-0.84	96[8]	N/A
Bi-Sn Aerogel	0.1 $KHCO_3$	-9.3	-1.0	93.9[9]	N/A
Ag/Sn-SnO_2	0.5 $KHCO_3$	-48.5	-1.37	90[10]	N/A
Sn-MOF/In_6	0.5 $KHCO_3$	-16.0	-0.96	97.5[11]	N/A

*Indicating this work and †indicating current density averaged over 48 hours.

Turnover frequency (TOF) calculation

The TOF for the catalyst over 24 hours was calculated using the following equation[12]:

$$TOF = \frac{Current \times Avogadro\ no.}{Faraday\ constant \times Number\ of\ Sn\ sites} \times time \qquad (S1)$$

The number of Sn active sites was calculated by integrating the charges under the oxidation peak of Sn^0 to Sn^{2+}.[13]

Peak area: 0.092289 *A V*

Charge: $Charge\ (C) = \frac{Peak\ area}{Scan\ rate} = \frac{0.092289\ A\ V}{0.01\ V\ s^{-1}} = 9.2289\ A\ s\ (C)$

Number of electrons: $No.electrons = \frac{Charge}{Charge\ of\ electron} = \frac{9.2289\ C}{1.60x10^{-19}\ C} = 5.76x10^{19}$

Number of Sn sites: $No.Sn\ sites = \frac{No.electrons}{electrons\ in\ Sn^{(0)}\ to\ Sn^{2+}} = \frac{5.76x10^{19}}{2} = 2.88x10^{19}$

TOF: $TOF = \frac{Current \times Avogadro\ no.}{Faraday\ const. \times No\ Sn\ sites.} \times time = \frac{(0.0436 \times 6.023x10^{23})}{(96485 \times 2.88x10^{19})} \times 3600^2 = 1.22x10^5\ h^{-1}$

References

(1) Deng, Y.; Zhao, J.; Wang, S.; Chen, R.; Ding, J.; Tsai, H.-J.; Zeng, W.-J.; Hung, S.-F.; Xu, W.; Wang, J.; Jaouen, F.; Li, X.; Huang, Y.; Liu, B. Operando Spectroscopic Analysis of Axial Oxygen-Coordinated Single-Sn-Atom Sites for Electrochemical CO2 Reduction. *J Am Chem Soc* 2023, *145* (13), 7242–7251. https://doi.org/10.1021/jacs.2c12952.

(2) Li, F.; Chen, L.; Knowles, G. P.; MacFarlane, D. R.; Zhang, J. Hierarchical Mesoporous SnO 2 Nanosheets on Carbon Cloth: A Robust and Flexible Electrocatalyst for CO 2 Reduction with High Efficiency and Selectivity. *Angewandte Chemie* 2017, *129* (2), 520–524. https://doi.org/10.1002/ange.201608279.

(3) Li, K.; Xu, J.; Zheng, T.; Yuan, Y.; Liu, S.; Shen, C.; Jiang, T.; Sun, J.; Liu, Z.; Xu, Y.; Chuai, M.; Xia, C.; Chen, W. In Situ Dynamic Construction of a Copper Tin Sulfide Catalyst for High-Performance Electrochemical CO2 Conversion to Formate. *ACS Catal* 2022, *12* (16), 9922–9932. https://doi.org/10.1021/acscatal.2c02627.

(4) Chen, G.; Ye, D.; Chen, R.; Li, J.; Zhu, X.; Liao, Q. Enhanced Efficiency for Carbon Dioxide Electroreduction to Formate by Electrodeposition Sn on Cu Nanowires. *Journal of CO2 Utilization* 2021, *44*, 101409. https://doi.org/https://doi.org/10.1016/j.jcou.2020.101409.

(5) An, X.; Li, S.; Yoshida, A.; Wang, Z.; Hao, X.; Abudula, A.; Guan, G. Electrodeposition of Tin-Based Electrocatalysts with Different Surface Tin Species Distributions for Electrochemical Reduction of CO2 to HCOOH. *ACS Sustain Chem Eng* 2019, *7* (10), 9360–9368. https://doi.org/10.1021/acssuschemeng.9b00515.

(6) Liu, S.; Pang, F.; Zhang, Q.; Guo, R.; Wang, Z.; Wang, Y.; Zhang, W.; Ou, J. Stable Nanoporous Sn/SnO2 Composites for Efficient Electroreduction of CO2 to Formate over Wide Potential Range. *Appl Mater Today* 2018, *13*, 135–143. https://doi.org/https://doi.org/10.1016/j.apmt.2018.08.014.

(7) Li, Z.; Cao, A.; Zheng, Q.; Fu, Y.; Wang, T.; Arul, K. T.; Chen, J.-L.; Yang, B.; Adli, N. M.; Lei, L.; Dong, C.-L.; Xiao, J.; Wu, G.; Hou, Y. Elucidation of the Synergistic Effect of Dopants and Vacancies on Promoted Selectivity for CO2 Electroreduction to Formate. *Advanced Materials* 2021, *33* (2), 2005113. https://doi.org/https://doi.org/10.1002/adma.202005113.

(8) Ren, B.; Wen, G.; Gao, R.; Luo, D.; Zhang, Z.; Qiu, W.; Ma, Q.; Wang, X.; Cui, Y.; Ricardez–Sandoval, L.; Yu, A.; Chen, Z. Nano-Crumples Induced Sn-Bi Bimetallic Interface Pattern with Moderate Electron Bank for Highly Efficient CO2 Electroreduction. *Nat Commun* 2022, *13* (1), 2486. https://doi.org/10.1038/s41467-022-29861-w.

(9) Wu, Z.; Wu, H.; Cai, W.; Wen, Z.; Jia, B.; Wang, L.; Jin, W.; Ma, T. Engineering Bismuth–Tin Interface in Bimetallic Aerogel with a 3D Porous Structure for Highly Selective Electrocatalytic CO2 Reduction to HCOOH. *Angewandte*

Chemie International Edition 2021, *60* (22), 12554–12559. https://doi.org/https://doi.org/10.1002/anie.202102832.

(10) Zhang, M.; Cao, A.; Xiang, Y.; Ban, C.; Han, G.; Ding, J.; Gan, L.-Y.; Zhou, X. Strongly Coupled Ag/Sn–SnO2 Nanosheets Toward CO2 Electroreduction to Pure HCOOH Solutions at Ampere-Level Current. *Nanomicro Lett* 2023, *16* (1), 50. https://doi.org/10.1007/s40820-023-01264-6.

(11) Yan, J.; Wang, X.; Ning, F.; Yi, J.; Liu, Y.; Wu, K. In-Modified Sn-MOFs with High Catalytic Performance in Formate Electrosynthesis from Aqueous Carbon Dioxide. *Dalton Transactions* 2023, *52* (34), 11904–11912. https://doi.org/10.1039/D3DT01610B.

(12) Burwell, T.; Thangamuthu, M.; Aliev, G. N.; Ghaderzadeh, S.; Kohlrausch, E. C.; Chen, Y.; Theis, W.; Norman, L. T.; Fernandes, J. A.; Besley, E.; Licence, P.; Khlobystov, A. N. Direct Formation of Copper Nanoparticles from Atoms at Graphitic Step Edges Lowers Overpotential and Improves Selectivity of Electrocatalytic CO2 Reduction. *Commun Chem* 2024, *7* (1), 140. https://doi.org/10.1038/s42004-024-01218-y.

(13) Anantharaj, S.; Kundu, S. Do the Evaluation Parameters Reflect Intrinsic Activity of Electrocatalysts in Electrochemical Water Splitting? *ACS Energy Lett* 2019, *4* (6), 1260–1264. https://doi.org/10.1021/acsenergylett.9b00686.
